# Omega-3 Recovers Cannabinoid 1 Receptor Expression in the Adult Mouse Brain after Adolescent Binge Drinking

**DOI:** 10.3390/ijms242417316

**Published:** 2023-12-10

**Authors:** Ane Martín-Llorente, Maitane Serrano, Itziar Bonilla-Del Río, Leire Lekunberri, Garazi Ocerin, Nagore Puente, Almudena Ramos, Irantzu Rico-Barrio, Inmaculada Gerrikagoitia, Pedro Grandes

**Affiliations:** 1 Laboratory of Ultrastructural and Functional Neuroanatomy of the Synapse, Department of Neurosciences, Faculty of Medicine and Nursing, University of the Basque Country UPV/EHU, 48940 Leioa, Spain; anemartinllorente@gmail.com (A.M.-L.); maitane.serrano@ehu.eus (M.S.); itziar.bonilla@ehu.eus (I.B.-D.R.); leire.lecumberri@ehu.eus (L.L.); garaziocerin97@gmail.com (G.O.); nagore.puente@ehu.eus (N.P.); almudena.ramos@ehu.eus (A.R.); irantzu.rico@ehu.eus (I.R.-B.); 2 Achucarro Basque Center for Neuroscience, Science Park of the UPV/EHU, 48940 Leioa, Spain

**Keywords:** ethanol, polyunsaturated fatty acids, endocannabinoid system, immunohistochemistry, motor system, cognition, rodent

## Abstract

Adolescent binge drinking is a social problem with a long-lasting impact on cognitive functions. The cannabinoid type-1 (CB_1_) receptor of the endocannabinoid system (ECS) is involved in brain synaptic plasticity, cognition and behavior via receptor localization at specific subcellular compartments of the cortical, limbic and motor regions. Alcohol (EtOH) intake affects the ECS, CB_1_ and their functions. Evidence indicates that binge drinking during adolescence impairs memory via the abrogation of CB_1_-dependent synaptic plasticity in the hippocampus. However, the impact of EtOH consumption on global CB_1_ receptor expression in the adult brain is unknown. We studied this using optical density analysis throughout brain regions processed for light microscopy (LM) immunohistotochemistry. CB_1_ staining decreased significantly in the secondary motor cortex, cerebellum, cingulate cortex, amygdala and nucleus accumbens. Next, as omega-3 (n-3) polyunsaturated fatty acids (PUFAs) rescue synaptic plasticity and improve EtOH-impaired cognition, we investigated whether docosahexaenoic acid (DHA) and eicosapentaenoic acid (EPA) had any effect on CB_1_ receptors. N-3 intake during EtOH abstinence restored CB_1_ immunostaining in the secondary motor cortex, cerebellum and amygdala, and ameliorated receptor density in the cingulate cortex. These results show that n-3 supplementation recovers CB_1_ receptor expression disrupted by EtOH in distinct brain regions involved in motor functions and cognition.

## 1. Introduction

The ECS system is mainly composed of endocannabinoids (eCBs), the enzymes involved in their synthesis and degradation, as well as cannabinoid receptors [1]. The cannabinoid CB_1_, the most abundant G-protein-coupled receptor in the brain, is highly expressed in the basal ganglia, cerebral cortex, cerebellum (Cb) and hippocampus, which is consistent with its role in learning and memory, motor control, motivation, reward and emotional processing, among many others [1,2]. Furthermore, CB_1_ increases and changes its distribution from adolescence to adulthood, is predominantly localized in presynaptic terminals and noticeably present in glial cells and mitochondria, modulates neurotransmitter release and also participates in synaptic plasticity [1,2,3,4,5].

Binge drinking during adolescence has a long-lasting impact on cognition and behavior via interference with brain neurochemical systems, including the ECS which participates in the regulation of motivation and EtOH intake [6,7,8,9]. EtOH exposure alters both CB_1_ mRNA and protein [10,11], changing the availability and binding of the receptor [12,13]. Conversely, gene deletion and CB_1_ receptor antagonism affect drinking behavior [14]. In addition, a CB_1_ mRNA decrease in the adult hippocampus after adolescent binge drinking correlates with CB_1_ receptor reduction in excitatory terminals of the hippocampal dentate molecular layer. These changes, together with the rise in monoacylglycerol lipase (MAGL) mRNA, the main degrading enzyme of the eCB 2-arachidonoylglycerol (2-AG), disrupt endocannabinoid-dependent long-term depression (LTD) and set off memory impairment [15]. Remarkably, the 2-AG increase elicited by MAGL inhibition recovers synaptic plasticity and cognition [15]. Although pharmacological manipulations are potential therapeutic strategies for treating alcohol-induced long-term cognitive deficits, nutritional supplementation might also stand up as a suitable alternative.

DHA and EPA are n-3 PUFAs that accumulate in brain cell membranes and maintain their structure and fluidity, restore glutathione levels, are anti-inflammatory, reduce oxidative stress and apoptosis, and overcome synaptic plasticity deficits, improving the cognitive impairment caused by prenatal EtOH exposure [16,17,18,19]. DHA is unevenly distributed among brain regions and between neurons and glial cells, and participates in membrane-associated protein functions, cellular signaling, gene expression, lipogenesis, neurogenesis and synaptic pruning [16]. EtOH decreases n-3 via different mechanisms [20,21]. The negative impact of EtOH on DHA alters synaptic plasticity in the hippocampus and medial prefrontal cortex, both enriched in DHA under normal conditions [16]. Noticeably, the enhancement of eCB signaling recovers emotional and cognitive functions as well as reverses the abrogated eCB-dependent synaptic plasticity caused by n-3 PUFA deficiency in brain regions processing mood and cognition [22,23,24]. Moreover, DHA modulates CB_1_ mRNA and protein expression [25] as well as eCBs [26,27]. In fact, a new class of n-3-derived eCBs has been identified in addition to the most-known n-6-derived 2-AG and anandamide (AEA) [28].

However, despite the close relationship between EtOH, n-3 and CB_1_ receptors, the direct impact of n-3 PUFAs on brain CB_1_ receptor expression after EtOH intake remains unknown. In this investigation, we studied the effects of an n-3-enriched diet on CB_1_ receptor expression in the adult brain after binge drinking during adolescence. In particular, we analyzed CB_1_ receptor optical density in fourteen brain regions that are sensitive to EtOH damage and are known to express CB_1_ receptors.

## 2. Results

### N-3 and Brain CB_1_ Receptor Expression

The brain and cerebellar CB_1_ immunostaining patterns in H_2_O, EtOH, n-3-EtOH and n-3-H_2_O mice corresponded well with previous observations of the CB_1_ receptor distribution in the mouse brain and cerebellum. CB_1_ immunoreactive punctate appeared concentratedin certain brain regions as well as in some cerebral and cerebellar cortical layers. Thus, strong CB_1_ receptor immunoreactivity was observed in the granule cell layer of the olfactory bulb (OB) [29] and remarkable staining was also revealed in the striatum, cerebral cortex (layers II–III and V–VI), olfactory tubercle, substantia nigra pars reticulata (SN), amygdala (Amg) and hippocampus [30], as well as the cerebellar molecular and Purkinje cell layers. More moderate immunoreactivity was detected in the nucleus accumbens (Acb) [31].

**Figure 1:**
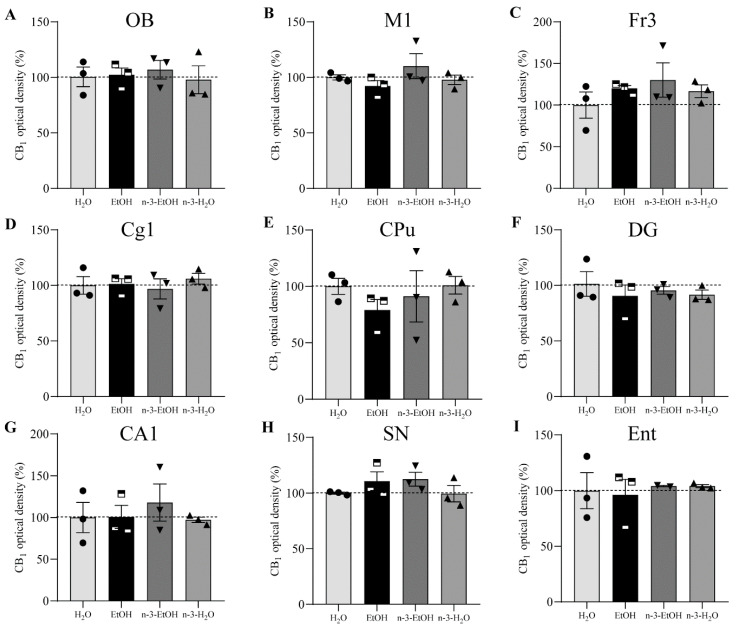
Normalized (%) CB_1_ receptor optical density in the OB (**A**), M1 (**B**), Fr3 (**C**), Cg1 (**D**), CPu (**E**), DG (**F**), CA1 (**G**), SN (**H**), and Ent (**I**). Pooled data are expressed as mean ± SEM (one-way ANOVA, Dunn’s and Holm Sidak’s multiple comparisons tests).

**Figure 2:**
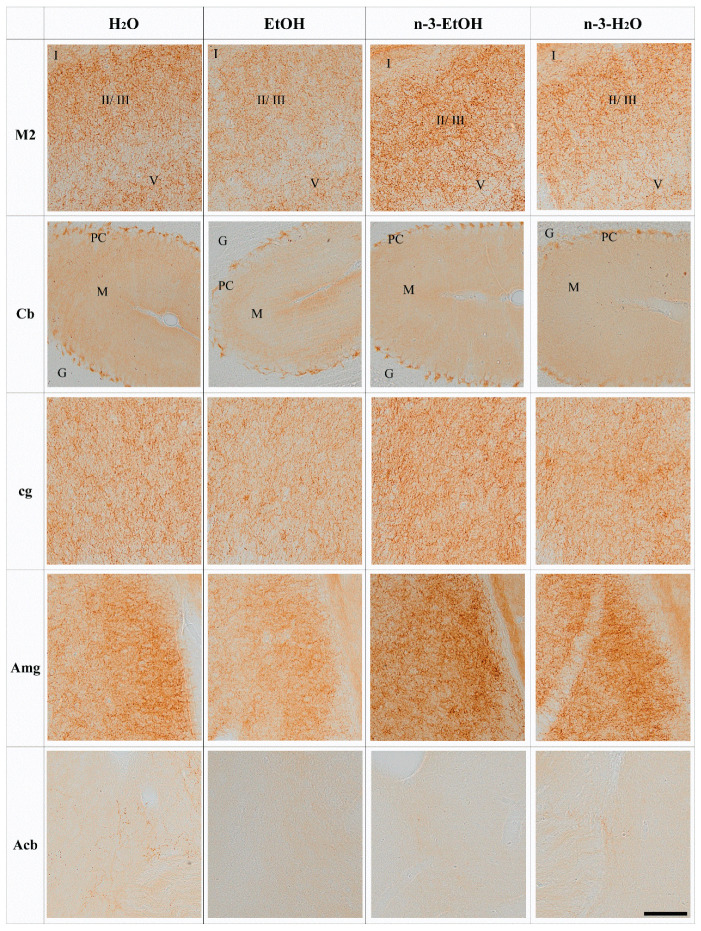
CB_1_ receptor-like immunoreactivity in the M2, Cb, cg, Amg and Acb of adult male mice exposed to H_2_O, EtOH, n-3-EtOH or n-3-H_2_O. Pre-embedding immunoperoxidase method for light microscopy. The typical CB_1_ staining pattern is observed: abundant dotty elements distributed in the superficial (II–III) and deep (V) layers of the M2 cortex as well as in the cingulum (cg) and amygdala (Amg); uniform immunostaining in the cerebellar molecular (M) layer, strong basket cell terminal labeling around Purkinje cell (PC) bodies in the PC layer, and lack of staining in the granule (G) cell layer; very faint staining in the Acb with only some positive varicose fibers in control (H_2_O). Scale bar: 200 µm.

**Figure 3:**
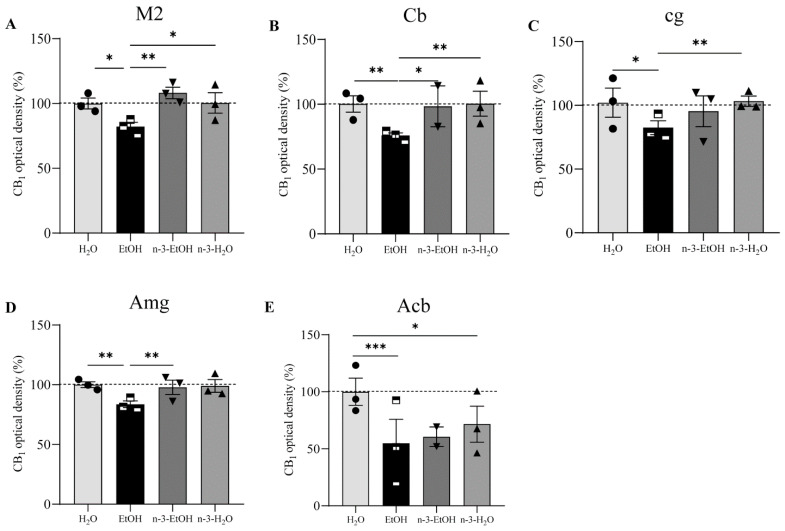
CB_1_ receptor optical density in adult male mice exposed to H_2_O, EtOH, n-3-EtOH or n-3-H_2_O. Normalized (%) CB_1_ optical density in the M2 (**A**), Cb (**B**), cg (**C**), Amg (**D**) and Acb (**E**). Pooled data are expressed as mean ± SEM (one-way ANOVA, Dunn’s multiple comparisons test; * *p* < 0.05; ** *p* < 0.01; *** *p* < 0.001). The n-3 diet during withdrawal recovers the significant decrease in CB_1_ receptor expression in the M2, Cb and Amg of the adult brain after adolescent binge drinking.

The analysis of the CB_1_ receptor optical density showed that the receptor distribution pattern varied in some regions depending on EtOH and/or n-3 intake. However, no changes were found in the OB, primary motor cortex (M1), frontal cortex (Fr3), cingular cortex area (Cg1), caudate putamen (CPu), dentate gyrus (DG), hippocampal CA1, SN and entorhinal cortex (Ent) in the four conditions (*p* > 0.05; Figure 1, Table 1). In contrast, the secondary motor cortex (M2), Cb, cingulum (cg), Amg and Acb were affected by EtOH and n-3 intake (*p* < 0.05; Figure 2). Thus, a significant reduction in CB_1_ receptor staining (~20%) was detected in the adult M2 after adolescent EtOH intake relative to the control (EtOH: 82.20 ± 3.732%; H_2_O: 100.00 ± 3.507% * *p* < 0.05; Figure 2 and Figure 3 A; Table 1). Remarkably, n-3 supplementation during withdrawal recovered CB_1_ receptor expression in the M2 to control levels (n-3-EtOH: 106.30 ± 4.604% ** *p* < 0.01), without the enriched diet having any effect on H_2_O mice (n-3-H_2_O: 100.50 ± 5.218% * *p* < 0.05; Figure 2 and Figure 3A; Table 1). The decrease in CB_1_ optical density following EtOH exposure during adolescence was more conspicuous in the Cb (EtOH: 76.40 ± 4.445% versus H_2_O: 100.00 ± 5.267%, ** *p* < 0.01), particularly in the molecular layer (Figure 2 and Figure 3B; Table 1). The n-3 diet normalized the detrimental effect of EtOH on CB_1_ values (n-3-EtOH: 98.43 ± 5.627%, * *p* < 0.05; Figure 2 and Figure 3B; Table 1). Again, n-3 under standard H_2_O conditions did not modify receptor expression in this region (n-3-H_2_O: 100.40 ± 4.620%, ** *p* < 0.01; Figure 2 and Figure 3B; Table 1). A similar effect of EtOH was observed in the cg, where CB_1_ optical density decreased significantly in adult mice after binge drinking during adolescence (EtOH: 80.77 ± 4.864% versus H_2_O: 100.00 ± 4.868% * *p* < 0.05; Figure 2 and Figure 3C; Table 1). However, n-3 was unable to revert significantly the receptor deficit (n-3-EtOH: 97.86 ± 5.274%), in contrast to the dietary effect on H_2_O (n-3-H_2_O: 105.20 ± 4.610%) relative to the CB_1_ decrease in EtOH mice (** *p* < 0.001; Figure 2 and Figure 3C; Table 1). The values in the Amg were also affected (Figure 2 and Figure 3D; Table 1): the significant CB_1_ reduction in EtOH mice (EtOH: 82.80 ± 3.468% versus H_2_O: 100.00 ± 3.594%, ** *p* < 0.01) returned to normal with n-3 intake (n-3-EtOH: 102.30 ± 3.977%, ** *p* < 0.01), with no effect of the nutritional supplementation when mice only drank H_2_O (97.69 ± 5.744%). Finally, CB_1_ optical density was drastically reduced in the Acb of the mature brain exposed to EtOH during adolescence (EtOH: 54.18 ± 10.81% versus H_2_O: 100.00 ± 6.028, *** *p* < 0.001; Figure 2 and Figure 3E; Table 1). However, n-3 intake could not restore CB_1_ receptor expression in EtOH mice (n-3-EtOH: 60.61 ± 8.153%). In addition, and contrary to the other studied brain regions, n-3 downregulated CB_1_ staining in H_2_O mice (n-3-H_2_O: 66.15 ± 11.04%, * *p* < 0.05; Figure 2 and Figure 3E; Table 1).

## 3. Discussion

### 3.1. Long-Lasting Effect of Adolescent Binge Drinking on CB_1_ Receptor Expression

We have shown that binge drinking during adolescence reduces CB_1_ receptor immunostaining in certain regions of the mature mouse brain, in particular, the M2, Cb, cg, Amg and Acb. Interestingly, n-3 supplementation during abstinence restores CB_1_ receptor expression measured using optical density in the M2, Cb and Amg and ameliorates density levels in the cg.

The CB_1_ receptor expression pattern matched the brain receptor distribution in the cortical, limbic and motor regions [2,30]. However, long-term changes in CB_1_ immunostaining after adolescent alcohol intake were restricted to some brain regions and the Cb that seem to correlate with the impact of EtOH intake on brain structure and function [6,32,33]. EtOH alters grey matter throughout the cortex, including the olfactory areas, Amg and Cb [33,34,35]. Also, the mesocorticolimbic system is affected. We observed that the long-lasting decrease in CB_1_ receptors normally expressed in the Acb [36] did not recover over time after adolescent binge drinking, remaining low even when the animals were under n-3 supplementation. As CB_1_ receptors intervene in brain maturation, it is plausible that the CB_1_ receptor expression deficits revealed in the Acb negatively contribute to the shape of the mesocorticolimbic system during the adolescent period, ultimately promoting brain vulnerability and alcohol addiction [35]. 

This study was conducted using male mice. Though males might be more vulnerable to withdrawal [37], females are more sensitive to EtOH [38,39]. Also, the effectiveness of treatments differs between males and females [40]. It is plausible that the EtOH impact on CB_1_ receptor expression and the effects of the n-3-enriched diet may vary between males and females, a possibility that will be explored in our future investigations.

The LM immunohistochemistry applied in the present study has some obvious limitations that deserve attention. Immunohistochemical techniques for LM were used in the 1990s to describe the pattern of CB_1_ receptor-like immunoreactivity in the brain [41,42]. Consequently, the strong immunostaining observed in certain brain regions (motor, limbic, reward, cortical) endorsed the advance in the knowledge of CB_1_ receptor functions in brain circuits. However, low CB_1_ receptor expression in cell types cannot be visualized using LM immunohistochemistry [30]. In addition, the tendency to diffusion of the 3-3′diaminobenzidine (DAB) reaction product used as chromogen in LM immunohistochemistry could lead to potential unspecific staining or false positives due to endogenous biotinylated proteins. These pitfalls can only be ruled out by using appropriate controls. In this study, we have used CB_1_-knockout brain tissue to discard bias, confirming the specificity of the CB_1_ staining observed throughout the brain in our experimental conditions. Ultimately, high-resolution immunoelectron microscopy that has been shown to be an excellent tool for unveiling the precise subcellular localization of CB_1_ receptors in the brain, would definitely identify the subcellular compartments and the CB_1_ receptor pools that were conspicuously reduced by adolescent EtOH intake, and recovered by n-3 in the specific brain regions identified in this study. 

Endocannabinoid levels, membrane fluidity and EtOH-degrading enzymatic machinery could contribute to the altered CB_1_ receptor pattern observed in the adult brain after adolescent binge drinking. In addition, EtOH intake during adolescence causes memory impairment that can last into adulthood [43,44]. Our model of binge drinking during adolescence used in this study has previously been shown to be associated with hippocampal memory deficits in adulthood [15,45]. Although CB_1_ receptor optical density was not significantly affected by binge drinking, subtle subcellular changes in receptor expression were detected in the dentate molecular layer that should contribute to the abrogation of cannabinoid-dependent synaptic plasticity at the excitatory medial perforant path synapses and related memory loss [15]. Remarkably, the deleterious cognitive binge drinking effects were recovered by increasing the endocannabinoid 2-AG or by environmental conditions [15,45]. Furthermore, the M2 and Cb, both affected by EtOH, are brain regions involved in motor coordination [46,47] and EtOH intake leads to motor incoordination and ataxia [43]. Our present results show a significant decrease in CB_1_ staining in the cerebellar molecular layer, where the receptor is mostly localized to the excitatory granule cell parallel fiber terminals [48]. However, the lack of CB_1_ receptors does not seem to cause evident cerebellar motor deficits [49], despite their role in motor learning [50]. Nevertheless, cannabinoid-dependent motor control is also exerted from the cortex [51], where we detected deficits in CB_1_ receptors upon adolescent binge drinking. Interestingly, young adult mice under enriched environment recovered motor coordination and balance after adolescent binge drinking [45]. It is likely that the ECS participates in this motor improvement, as it is the case in the memory recovery elicited by environmental enrichment via the restoration of endocannabinoid-dependent excitatory synaptic plasticity, in which CB_1_ receptors, group I metabotropic glutamate receptors and 2-AG were involved [9].

EtOH modifies synaptic membrane fluidity [52] and stimulates arachidonic acid (AA) production from membrane phospholipids by increasing phospholipase A_2_ (PLA_2_) [53]. The availability of more AA for AEA synthesis may be responsible for the decrease in CB_1_ agonist binding and gene expression elicited by chronic EtOH in certain brain regions [54,55]). In fact, the drop in CB_1_ receptor immunostaining in the cerebellar molecular layer correlates with AEA transport inhibition and a 2-AG increase in granule cells after chronic EtOH [56,57]. Cannabinoids internalize CB_1_ receptors and reduce their mobility, having an impact on receptor availability at the synapse [58]. We have demonstrated previously that Δ-9-tetrahydrocannabinol (THC) causes a selective CB_1_ receptor labeling decrease in certain subcellular compartments (excitatory and inhibitory terminals, mitochondria, astrocytes) of several brain regions [59]. This distinct impact of THC could be related to the different THC levels and metabolites detected among brain regions after acute THC administration [60]. A similar phenomenon could befall our model of binge drinking. In fact, brain EtOH metabolism by class III alcohol dehydrogenase (ADH) generates acetaldehyde that accumulates in the hippocampus, cortex and Cb, where the enzyme is more expressed [61]. Interestingly, the enzyme distribution coincides in brain regions with high CB_1_ receptor expression, such as the cortex and Cb, both strikingly affected by adolescent binge drinking. EtOH decreases glutathione in these same regions, thus increasing oxidative processes and brain damage in a model of prenatal EtOH exposure [17]. As class III ADH is a glutathione-dependent formaldehyde dehydrogenase, it is likely that the glutathione reduction elicited by EtOH and the consequent oxidative state leads to enzyme malfunction, jeopardizing EtOH elimination in regions where the enzyme is more abundant.

### 3.2. N-3 Recovers CB_1_ Receptor Expression in the Brain

DHA and AA are major phospholipid components of brain cell membranes [16,19]. EtOH reduces DHA in the brain [20,21] and its deficit impacts on both cell membranes, altering their biophysical properties, and related membrane proteins, such as enzymes and receptors [19]. N-3 deficiency lowers CB_1_ receptors in different brain regions [62] and impairs endocannabinoid-mediated synaptic plasticity [22]. The Fr3, OB, Cb, hippocampus, midbrain and striatum rank in high-to-low order among the brain regions with more DHA [63]. However, the negative effect on CB_1_ receptor expression in the adult brain after adolescent binge drinking was particularly outstanding in the M2, Cb and Acb (ventral striatum), with no effect in the hippocampus, dorsal striatum, some cortical areas, OB and SN. A DHA-enriched diet counteracts the low brain n-3 PUFA levels due to EtOH intake [21] and reverses EtOH-induced impairment of synaptic plasticity [18]. It also restores aquaporin-4, PLA-2 and glutathione affected by EtOH [17,21,64,65]. The recovery of CB_1_ receptor immunostaining by n-3 supplementation during the abstinence period points to the normalization of cell membrane homeostasis. 

In conclusion, abusive EtOH consumption during adolescence alters CB_1_ receptor immunostaining optical density in some brain regions of the adult mouse, and an n-3-enriched diet recovers the reduced CB_1_ expression in limbic and motor structures following binge drinking. Uncovering the PUFA effects and mechanisms by which the n-3-enriched diet can impact on brain cannabinoid receptor expression (as shown in this paper) and function after adolescent binge drinking, could be an appropriate non-pharmacological approach to counteract the EtOH impact on cannabinoid-dependent synaptic plasticity, cognition and behavior. 

## 4. Material & Methods

### 4.1. Generation of CB_1_-KO

CB_1_-knockout (CB_1_-KO) mice were generated and genotyped as previously described [66] and formerly collected [30]. They were obtained by crossing CB_1_^f/f^ mice with CMV-Cre mice (“Cre deleter”). Mice were of a predominant C57BL/6-N background (9–10 back-crossings) and the breeding strategy used was female CB_1_^+/−^ × male CB_1_^+/−^ (Table 2).

### 4.2. Animal Treatment

**Figure 4:**
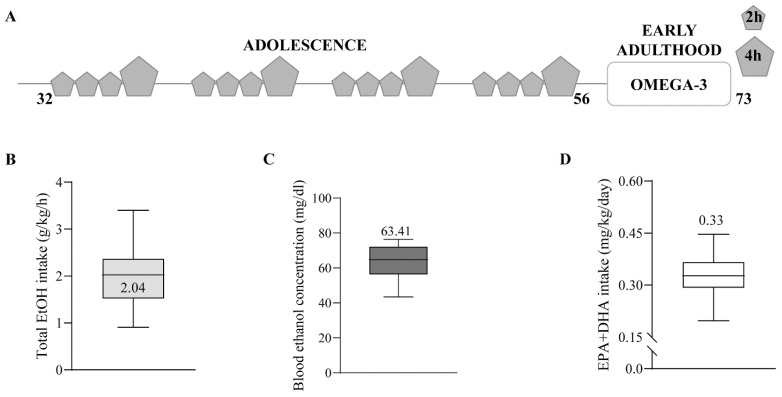
Schematic timeline of the EtOH procedure, total EtOH intake, BEC, EPA and DHA intake. (**A**) C57BL/6J male mice were submitted to the DID procedure over four weeks (PND 32–56). They had 2 h free access to H_2_O or EtOH for the first three days of the week, and 4 h the fourth day. During abstinence (PND 56–73), half of them were fed with n-3 supplementation (EPA and DHA 2%). (**B**) Average of total EtOH intake during DID of EtOH (2.033 ± 0.5437 g/kg/h, n = 8) and n-3-EtOH (2.051 ± 0.5941 g/kg/h, n = 8) (Student’s *t*-test, *p* > 0.05). (**C**) Average BEC obtained on the last day of EtOH exposure in EtOH (62.96 ± 10.89 mg/dL; n = 8) and n-3-EtOH (63.92 ± 10.08 mg/dL; n = 7) (Student’s *t*-test, *p* > 0.05). (**D**) Average of total EPA and DHA intake during withdrawal in n-3-H_2_O (0.332 ± 0.0727 mg/kg/day, n = 12) and n-3-EtOH mice (0.324 ± 0.0679 mg/kg/day, n = 12) (Student’s *t*-test, *p* > 0.05).

Four-week-old C57BL/6J male mice (Janvier Labs, Le Genest-Saint-Isle, France) were housed in pairs and habituated to a dark cycle (8 a.m.–8 p.m.). Then, they were exposed to water or drinking-in-the-dark (DID) during adolescence, as previously described [45]. Briefly, the mice were individualized and exposed to a bottle of 10 mL tap water or EtOH 20% (Boter S.L., Barcelona, Spain) four days a week during four weeks (postnatal day (PND) 32 to 56). They had free access to the bottle for 2 h the first three days, and 4 h the fourth day. Mice were resting and kept in pairs with food and water ad libitum for the last three days of the week (Figure 4A–C). On PND 56, a blood sample was collected from the lateral tail vein using a capillary tube 30 min after 4 h of EtOH exposure (Sarstedt, Nümbrecht, Germany). Blood samples were analyzed for EtOH concentration using a commercial OH assay kit (Abcam ab65646, Madrid, Spain), following manufacturer instructions. Half of the mice were fed 2% EPA and DHA (2.2% EPA and 1.5% DHA of total fats; SAFE, Augy, France) during withdrawal (PND 56–73). Twice a week, mice and food were weighed to measure EPA and DHA intake (mg/kg/day) (Figure 4D). Three mice/group were culled on PND 73. They were deeply anesthetized using 4% chloral hydrate (10 mL/kg body weight, i.p.) and perfused through the left ventricle with 30 mL phosphate-buffered saline (PBS, 0.1 M, pH 7.4), followed by 80 mL of the fixative (4% formaldehyde depolymerized from paraformaldehyde, 0.2% picric acid, 0.1% glutaraldehyde) prepared in PBS at room temperature (RT). Brains were removed and post-fixed as described elsewhere in detail [59].

### 4.3. Antibody Characterization

**Figure 5:**
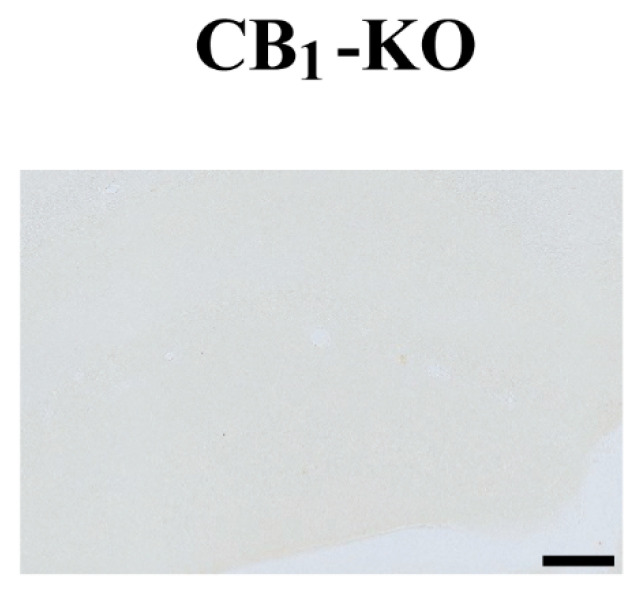
Specificity of the CB_1_ antibody tested in brain tissue (hippocampus) lacking CB_1_ receptors (CB_1_-KO). Pre-embedding immunoperoxidase method for light microscopy. No trace of staining canbe detected. Scale bar: 200 µm.

The specificity of the CB_1_ receptor antibody (Nittobo Medical Co., Ltd., Tokyo, Japan); goat polyclonal, CB_1_-Go-Af450, RRID AB_2571592, Table 3) has been tested thoroughly [3,4,30]. In addition, CB_1_ receptor staining was not detected in the CB_1_-KO mouse brain (Figure 5).

### 4.4. Immunohistochemistry for Light Microscopy

This was performed following the protocol previously published [30]. Briefly, coronal and sagittal sections cut at 50 μm on a vibratome (Leica VT 1000s, Wetzlar, Germany) were taken rostro-caudally from the whole brain and Cb, respectively, and collected in phosphate buffer (PB 0.1 M, pH 7.4) at RT. They were pre-incubated in a blocking solution for 30 min at RT, and then incubated in goat anti-CB_1_ receptor antibody (2 μg/mL) diluted in 10% horse serum, 0.1% sodium azide, 0.5% Triton in 1× Tris-HCl-buffered saline (TBS) overnight at RT. After washing in 1% horse serum and 0.5% Triton in 1× TBS the next day, tissue sections were incubated with a horse anti-goat IgG biotinylated antibody (H + L) (1:200, Vector Labs, Newark, CA, USA, cat#BA9500; RRID: AB_2336123) for 1 h at RT. Following several washes, they were incubated in avidin-biotin peroxidase complex (1:50, Vector Labs, Newark, CA, USA, Cat#PK-6100, RRID: AB_2336819) for 1 h at RT. Tissue was washed several times with the last two containing 0.5% Triton in PB, and incubated in 0.05% DAB (Sigma-Aldrich, Merck KGaA, Darmstadt, Germany, Cat#D5637; RRID: AB_2336819) with 0.01% hydrogen peroxide prepared in 0.1 M PB for 3 min. Finally, following five washes with 0.5% Triton in PB, the sections were mounted, dehydrated and coverslipped with DPX (Sigma Aldrich, Merck KGaA, Darmstadt, Germany Cat#44581).

### 4.5. Semiquantitative Analysis of CB_1_ Receptor Optical Density

Brain and cortical regions known to express CB_1_ receptors were selected: OB, M1, M2, Fr3, Cg1, cg, CPu, Acb, Amg, DG, hippocampal CA1, SN, Ent and Cb. Micrographs were taken at 10× using a light microscope (Olympus BX61, Hamburg, Germany) and processed using the Olympus cellSens Dimension using consecutive sections containing the brain regions and cortical areas of interest. For each region, three independent optical density measurements were performed at 10×, and two more were taken in a blank zone to rank background level. As some regions were through several slides, they were analyzed repeatedly in each mouse of the four experimental conditions (Table 4). All measurements were then pooled by mouse. Subsequently, data were normalized to 100% of the H_2_O group. Image J software (1.8.0_322); NIH; RRID:SCR_003070) and a statistical software package were used (GraphPad Prism 8; RRID: SCR_002798). The Shapiro–Wilk normality test was applied before running one-way ANOVA. Parametric data were analyzed using Holm Sidak’s multiple comparison test and non-parametric data using Dunn’s multiple comparison test. All values are given as mean ± SEM.

## Figures and Tables

**Table 1:** Normalized values (% mean ± SEM) of CB_1_ receptor optical density in olfactory bulb (OB), primary and secondary motor cortex (M1, M2), frontal cortex (Fr3), cingular cortex area 1 (Cg1), cingulum (cg), caudate putamen (CPu), nucleus accumbens (Acb), amygdala (Amg), dentate gyrus (DG), hippocampal CA1, substantia nigra (SN), entorhinal cortex (Ent) and cerebellum (Cb) for each experimental condition (n = 3 mice/group).

	H_2_O	EtOH	n-3-EtOH	n-3-H_2_O
OB	100.00 ± 3.982	102.30 ± 5.493	108.50 ± 4.499	100.40 ± 6.144
M1	100.00 ± 4.074	89.57 ± 4.427	106.80 ± 6.384	92.25 ± 5.785
M2	100.00 ± 3.507	82.20 ± 3.732	106.30 ± 4.604	100.50 ± 5.218
Fr3	100.00 ± 15.57	114.10 ± 14.25	130.20 ± 12.08	116.60 ± 13.00
Cg1	100.00 ± 6.694	77.99 ± 9.688	77.06 ± 14.08	90.67 ± 10.22
cg	100.00 ± 4.868	80.77 ± 4.864	97.86 ± 5.274	105.20 ± 4.610
CPu	100.00 ± 11.31	79.01 ± 11.61	83.25 ± 12.03	90.02 ± 15.31
Acb	100.00 ± 6.028	54.18 ± 10.81	60.61 ± 8.153	66.15 ± 11.04
Amg	100.00 ± 3.594	82.80 ± 3.468	102.30 ± 3.977	97.69 ± 5.744
DG	100.00 ± 7.651	92.42 ± 4.135	96.72 ± 4.383	93.04 ± 3.291
CA1	100.00 ± 10.62	100.80 ± 10.23	103.80 ± 5.839	98.02 ± 3.491
SN	100.00 ± 4.788	111.20 ± 3.457	109.10 ± 3.957	99.57 ± 3.918
Ent	100.00 ± 8.555	96.17 ± 4.879	104.20 ± 7.367	103.70 ± 3.471
Cb	100.00 ± 5.267	76.40 ± 4.445	98.43 ± 5.627	100.40 ± 4.620

**Table 2:** CB_1_-knockout mice.

**Name**	**Mouse Line Derived from**	**Background**	**Breeding Strategy Used**
CB_1_-KO	CB_1_-KO (CB_1_^−/−^) Originally obtained by crossing CB1^f/f^ mice with CMV-Cre mice (“Cre deleter”) [66]	Predominant C57BL/6-N (9–10 back-crossings)	Female CB_1_^+/−^ X Male CB_1_^+/−^

CB_1_, Type-1 cannabinoid; CB_1_-KO, Cannabinoid type-1 receptor knockout mouse.

**Table 3:** Primary antibody used for immunohistochemistry.

Antibody	Immunogen	Manufacturer, Species, Catalog Number, Rrid	Dilution	Characterization
ANTI-CB1	Recognizes the last 31 amino acids of the C-terminus of the mouse CB_1_ receptor (NM007726), as provided by the manufacturer: NCBI Reference Sequence: NP_031752.1; 443–473 amino acid residues: MHRAAESCIKSTVKIAKVTMSVSTDTSAEAL	Frontier Institute; Goat polyclonal; #CB_1_-Go-Af450, RRID: AB_2571592	2 µg/mL	On immunoblot, the antibody detects a single protein band at 52 kDa

**Table 4:** Number of measurements taken in the olfactory bulb (OB), primary and secondary motor cortex (M1, M2), frontal cortex (Fr3), cingular cortex area 1 (Cg1), cingulum (cg), caudate putamen (CPu), nucleus accumbens (Acb), amygdala (Amg), dentate gyrus (DG), CA1 hippocampus, substantia nigra (SN), entorhinal cortex (Ent) and cerebellum (Cb) for each experimental condition (n = 3 mice/group).

	H_2_O	EtOH	n-3-EtOH	n-3-H_2_O
OB	39	54	45	30
M1	72f	69	48	66
M2	72	63	48	66
Fr3	18	18	18	18
Cg1	18	18	12	18
cg	102	105	84	70
CPu	18	18	15	18
Acb	18	18	12	18
Amg	90	75	54	60
DG	51	51	45	36
CA1	36	36	27	21
SN	51	51	42	36
Ent	36	36	21	24
Cb	45	30	24	36

## Data Availability

The data presented in this study are available on request from the corresponding author.
